# Electrochemical Preparation of Polyaniline Nanowires with the Used Electrolyte Solution Treated with the Extraction Process and Their Electrochemical Performance

**DOI:** 10.3390/nano8020103

**Published:** 2018-02-12

**Authors:** Ying Wu, Jixiao Wang, Bin Ou, Song Zhao, Zhi Wang, Shichang Wang

**Affiliations:** 1 Clean Energy Research Center, School of Chemical Engineering and Technology, Tianjin University, Tianjin 300354, China; wuying19901213@163.com (Y.W.); binou@tju.edu.cn (B.O.); zhaosong@tju.edu.cn (S.Z.); wangzhi@tju.edu.cn (Z.W.); jiswang@tju.edu.cn (S.W.); 2 Tianjin Key Laboratory of Membrane Science and Desalination Technology, Tianjin University, Tianjin 300354, China; 3 State Key Laboratory of Chemical Engineering, Tianjin University, Tianjin 300354, China; 4 Collaborative Innovation Center of Chemical Science and Engineering (Tianjin), Tianjin University, Tianjin 300354, China

**Keywords:** polyaniline nanowires, electrochemical polymerization, p-benzoquinone, supercapacitor, extraction

## Abstract

Electrochemical polymerization of aniline is one of the most promising methods to prepare polyaniline (PANI) materials. However, during this process, the electrolyte solution must be replaced after electropolymerization of a certain time because of the generation and the accumulation of the by-products, which have significant effects on the morphology, purity and properties of PANI products. Treatment and recycling of the used electrolyte solution are worthwhile to study to reduce the high treatment cost of the used electrolyte solution containing aniline and its polymerization by-products. Here, the composition of the used electrolyte solution was separated and determined by high performance liquid chromatography coupled with diode array detection (HPLC-DAD) in the range of ultraviolet and visible (UV-Vis) light. The analysis results revealed that the used electrolyte solution consisted of aniline, p-hydroquinone (HQ), p-benzoquinone (BQ), co-oligomers of aniline and p-benzoquinone (CAB) and acid. Then, n-octanol and 2-octanone were selected as extracts to remove HQ, BQ and CAB from the used electrolyte solution. Following that, the recycled electrolyte solution was prepared by adjusting the concentration of aniline and acid of the aqueous phase, and the electrochemical polymerization process was conducted. Finally, the obtained PANI was characterized by scanning electron microscope (SEM) and electrochemical methods. The experimental results clearly demonstrate that the morphology and specific capacitance of PANI produced from the recycled electrolyte solution can be recovered completely. This research paves the way for reusing the used electrolyte solution for aniline electrochemical polymerization.

## 1. Introduction

Polyaniline (PANI), especially its nanomaterials, has tremendous promising applications in sensors, actuators [1,2], supercapacitors [3], anti-corrosive coatings, storage batteries [4], and etc., due to its giant specific surface area, short diffusion route and perfect dispersity [5,6]. PANI can be prepared by chemical or electrochemical polymerization. The electrochemical polymerization process has some superior characteristics over the chemical process due to its high atomic efficiency, tunable potential applied patterns and windows and the simplicity of the used electrolyte solution. However, the electrochemical method also has disadvantages compared to the chemical method due to its low production yield, difficulty in being conducted at low temperature and difficulty in scale-up. High production yield means that long-term electropolymerization is needed, which will induce the accumulation of by-products. The by-products including p-hydroquinone (HQ), p-benzoquinone (BQ) and co-oligomers of aniline and p-benzoquinone (CAB) and acid have significant side effects on the morphology, purity and properties of PANI products. In order to obtain high quality PANI, the electrolyte solution must be replaced with a fresh one. The discarded electrolyte solution not only contains waste chemicals, but also heavily pollutes the environment. Therefore, treating and recycling of the used electrolyte solution generated during PANI preparation need to be better addressed, which is an important issue for the production of PANI or its nanomaterials at a large commercial scale.

It was reported that the waste water containing aniline and phenols can be treated by several techniques, including physical processes (adsorption [7], membrane separation [8], extraction), chemical processes (chemical oxidation [9,10,11], electrochemical oxidation [12,13], photo-catalyst oxidation [14]) and biological processes (biodegradation [15], enzyme catalysis [16]). However, the high acidity, the high concentration of protonatedaniline and its by-products make it difficult to treat the used solution with biological processes, and it can only be treated with physical or chemical processes, which are high-cost approaches. In order to delimitate the pollutants completely, powerful oxidation methods like the Fenton process are required [10,17]. That is to say, the direct oxidation treatment of the used solution will not only greatly increase the cost of waste treatment, but also damage the recyclable aniline and doping acid. Thus, how to selectively remove HQ, BQ and CAB from the used electrolyte solution is the key issue for recycling after electrochemical polymerization of PANI nanowires.

Aniline, a weak base, exists in two forms, neutral aniline and protonated aniline. Aniline is almost always in the protonated form under the condition of a pH value below 2 [18]. The polarity of protonated aniline is stronger than HQ, BQ and CAB, indicating that HQ, BQ and CAB would have better solubility in organic solvent. Thus, with the utilization of the dissolubility difference in aqueous and organic solvents, HQ, BQ and CAB can be selectively removed from the used electrolyte solution by an extraction process under a strong acidic condition. After the treatment, the used electrolyte solution could be recycled in the production of PANI nanowires.

In this paper, the composition of the used electrolyte solution was analyzed by HPLC-DAD and UV-Vis, and the by-reactions’ formation mechanism was discussed. The influence of by-products on the electropolymerization of PANI was also discussed. In order to remove the by-products from the used electrolyte solution, n-octanol and 2-octanone were used as the extraction solvent for the treatment of the used electrolyte solution. After that, the concentration of aniline in the recycled electrolyte solution was adjusted, and the electrochemical polymerization process was conducted. The experimental results demonstrate that the morphology and specific capacitance of PANI produced from the recycled electrolyte solution can be recovered completely. The PANI obtained by the polymerization in fresh electrolyte solution (P-Fresh) has a uniform nanowire shape and high specific capacitance (576.6 F/g). Due to the by-products, the morphology of PANI obtained by the polymerization in the used electrolyte solution changed into nanosheets, submicron rods and aggregated particles, and the specific capacitance was reduced to 457.5 F/g, which can be seen in the initial electropolymerization. PANI produced in the recycled electrolyte solution has a uniform nanowire shape and high specific capacitance (571.0 F/g), similar to P-Fresh.

## 2. Materials and Methods

### 2.1. Materials

Aniline monomer (analytical grade, Shanghai Aladdin Bio-Chem Technology Co., Ltd., Shanghai, China) was distilled under reduced pressure and stored in the dark in a refrigerator before use. Sulfuric acid (analytical grade, 98%), n-octanol (analytical grade), 2-octanone (analytical grade) and acetonitrile (ACN, HPLC grade) were purchased from Shanghai Aladdin Bio-Chem Technology Co., Ltd. The deionized water used in this work is 18 MΩ/cm quality water. High purity water was prepared by the Milli-Q water-purification system (Millipore, Billerica, MA, USA). Stainless steel (SS, 316) sheets were polished with 800# and then 2000# emery paper and washed thoroughly with deionized water until free of emery particles.

### 2.2. Methods

#### 2.2.1. Preparation of the Used Electrolyte Solution

**Scheme 1:**
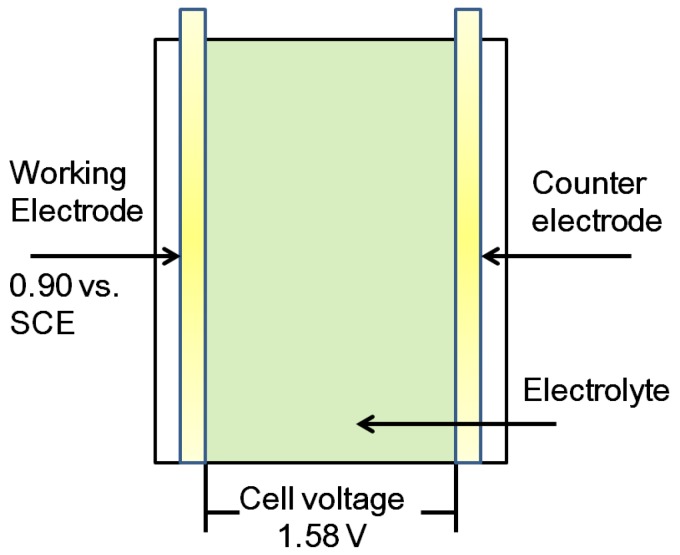
The diagram of the electrolytic cell. The distance between the working electrode and electrode was 1.0 cm, and the electrodes were SS (10.0 cm × 10.0 cm). The working electrode potential is 0.90 V vs. SCE, and the cell voltage is 1.58 V.

In this work, the fresh electrolyte solution was composed of 0.25 M aniline and 0.75 M sulfuric acid. The fresh electrolyte solution was added into the electrolytic cell, and the used electrolyte solution was obtained by collection of the electrolyte solution after electropolymerization. After n repetition of the electropolymerization of aniline for 20 min at 293 K, the used electrolyte solution was denoted as the n-th electrolyte solution (n = electropolymerization times). The cell voltage is 1.58 V, which is the potential difference between the working and counter electrode. The working electrode potential is 0.90 V vs. the saturated calomel electrode (SCE). After every electropolymerization reaction, the PANI products were brushed off the electrodes. The 10th electrolyte solution was prepared by addition of fresh electrolyte solution into the electrolytic cell and 10 repetitions of 20-min electropolymerization. The total electropolymerization time of the 10th electrolyte solution is 200 min. The diagram of the electrolytic cell can be seen in Scheme 1. The distance between the working electrode and counter electrode was 1.0 cm, and the electrodes were SS (10.0 cm × 10.0 cm).

#### 2.2.2. The Treatment and Recycling of the Used Electrolyte Solution

Forty milliliter of the 10th electrolyte solution were extracted with 4.0 mL extraction solvent, and the extraction was repeated 13 times. The extraction process was conducted as follows: The system was stirred 5 min and then left standing still until the clear phase interface formed. The organic phase and aqueous phase were collected separately. After 13 extractions, the aqueous phase extracted by n-octanol was denoted as NOCT, and the aqueous phase extracted by 2-octanone was denoted as 2OCT, respectively. A certain amount of aniline was added into NOCT and 2OCT in order to maintain their concentrations at 0.25 M, which are denoted as recycled electrolyze NOCT (RNOCT) and recycled electrolyze 2OCT (R2OCT).

#### 2.2.3. Preparation of PANI and PANI Electrode

PANI was prepared by the electropolymerization of aniline using a stainless steel electrode (2.0 × 3.0 cm) with an electrolysis potential of 0.90 V vs. SCE at 293 K for 20 min. The products were brushed off the electrodes, filtrated with membranes (pore diameter 0.22 μm) and washed with deionized water until the filter liquor was colorless, to collect PANI. After drying in a vacuum, the PANI was dispersed in distilled water. An amount of this PANI dispersion was deposited on Pt sheets (1.0 cm × 0.5 cm) and dried in a vacuum to be tested. The PANI obtained by electropolymerization in the fresh electrolyte solution and the 10th electrolyte solution was recorded as P-Fresh and P-10; the PANI obtained by electropolymerization in RNOCT and R2OCT was recorded as P-RNOCT and P-R2OCT, respectively; the PANI obtained by the fresh electrolyte solution with the addition of 0.001 M BQ, 0.005 M BQ and 0.01 M BQ was denoted as P-BQ1, P-BQ2 and P-BQ3, respectively.

#### 2.2.4. Characterization

The morphology of PANI was characterized by scanning electron microscopy (SEM, JSM6700F, JEOL Ltd., Tokyo, Japan). In SEM analysis, the PANI was deposited on a filter membrane by dropping the dispersion on the surface and drying in a vacuum. Gold sputtering was repeated 3 times at 10 mA for 15 s. The electrochemical behavior of PANI was characterized by the CV technique, and the CV was conducted in a three-electrode configuration using a Pt sheet coated with PANI as the working electrode, a Pt sheet as the counter electrode and a saturated calomel electrode (SCE) as the reference electrode at 25 mV/s. The electrolyte solution used in the experiments was a 1.0 M sulfuric acid solution deaerated by using high purity nitrogen gas at least 15 min before use. The specific capacitance of PANI was calculated from the CV curves according to the equation: [3]:

$$C_{m}=\frac{\int IdU}{mc\nu U}$$

where $C_{m}$ is the specific capacitance based on the mass of PANI in F/g, *I* is the response current in ampere (A), *U* is the scanned potential window in volt (V), ν is the potential scan rate in V/s, *m* is the mass of PANI dispersion on the electrode in gram (g) and *c* is the mass fraction of PANI in the PANI dispersion.The UV-Vis spectra of the used electrolyte solution were measured by a spectrophotometer (TU-1900, Beijing Purkinje General Instrument Co., Ltd., Beijing, China). The HPLC-DAD analysis was performed on a Waters Alliance 2695 HPLC system equipped with a Waters 2996 photodiode array detecto (Waters, Milford, MA, USA) and the Empower software package (Waters, Milford, MA, USA). The conditions of chromatography in HPLC-DAD analysis were: (1) the injection volume was 10 μL; (2) the composition of the mobile phase was high purity water/ACN = 45/55; (3) the flow rate of the mobile phase was 0.6 mL/min; (4) the analytical column (Symmetry Shield RP18.TM Column, 5 μm, 3.9 mm × 150 mm) was thermostated at 298 K; (5) the detection wavelength range was between 210 nm and 700 nm.

## 3. Results and Discussion

### 3.1. The Composition of the Used Electrolyte Solution

**Figure 1:**
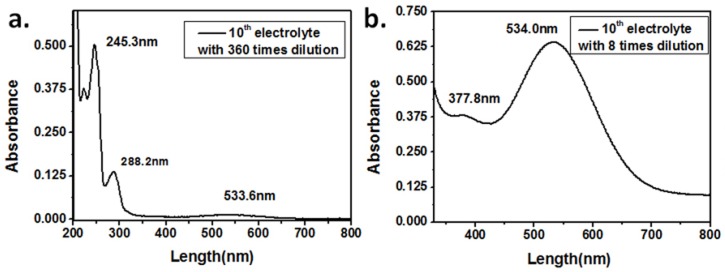
The UV-Vis spectrum of: (**a**) the 10th electrolyte solution with 360-times dilution; (**b**) the 10th electrolyte solution with eight-times dilution.

**Figure 2:**
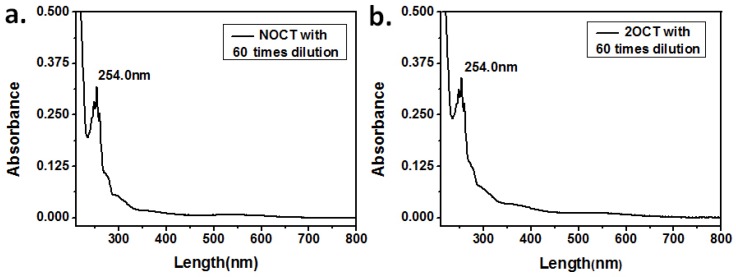
UV-Vis spectrum of the electrolyte solutions after the extraction treatment of the 10th electrolyte solution (**a**) n-octanol extracted aqueous phase (NOCT) with 60-times dilution; (**b**) 2-octanone extracted aqueous phase (2OCT) with 60-times dilution.

The 10th electrolyte solution is the used electrolyte solution obtained by 10 times of electropolymerization; its preparation process is referred to in Section 2.2.1. The UV-Vis spectra of the 10th electrolyte solution are shown in Figure 1. The curves appearing in Figure 1a,b clearly show that there are four absorption peaks centered at 245.3 nm, 288.2 nm, 377.8 nm and 534.0 nm, which were ascribed to the combination of the π-π* transition of aniline, BQ, HQ, co-oligomers of aniline and p-benzoquinone (CAB) [19,20] and the oxidized PPD-protonated *N*-phenyl-1,4-benzoquinone diimine [21,22], respectively. In this research, the 10th electrolyte solution was treated through an extraction process. After being extracted by n-octanol and 2-octanone, the UV-Vis spectra of the electrolyte solutions as shone in Figure 2a,b have only one absorption peaks centered at 254.0 nm, which was ascribed to the absorption of aniline’s π-π* transition, just as that of fresh electrolyte solution. The experimental results demonstrate that the by-products can be removed from the used electrolyte solution by extraction with n-octanol and 2-octanone.

**Figure 3:**
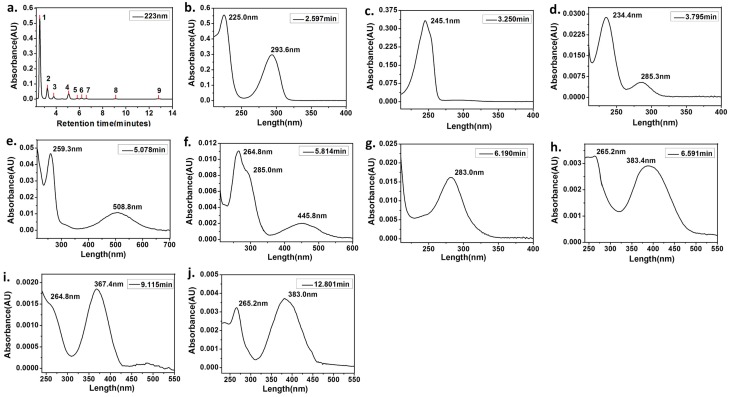
(**a**) The chromatograms of the n-octanol organic phase (ONOCT) in the extraction of the 10th electrolyte solution with the appropriate dilution degree at λ = 223 nm; the UV-Vis spectra of the peaks of ONOCT: (**b**) Peak 1, retention time (RT) = 2.597 min; (**c**) Peak 2, RT = 3.250 min; (**d**) Peak 3, RT = 3.795 min; (**e**) Peak 4, RT = 5.078 min; (**f**) Peak 5, RT = 5.814 min; (**g**) Peak 6, RT = 6.190 min; (**h**) Peak 7, RT = 6.591 min; (**i**) Peak 8, RT = 9.115 min; (**j**) Peak 9, RT = 12.901 min.

**Figure 4:**
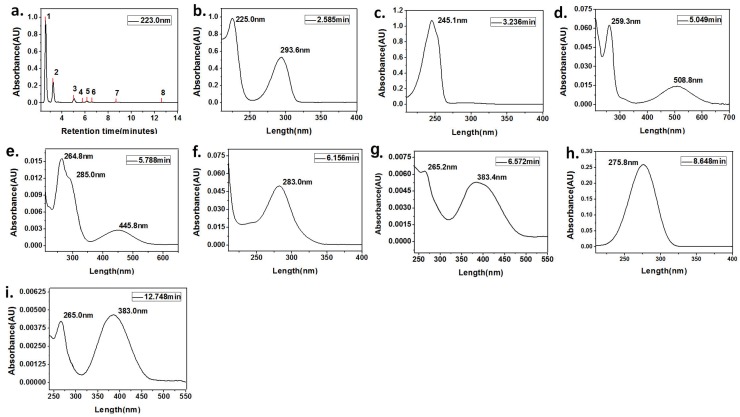
(**a**) The chromatograms of the 2-octanone organic phase (O2OCT) in the extraction of the 10th electrolyte solution with appropriate dilution degree at λ = 223 nm; the UV-Vis spectrums of the peaks of O2OCT: (**b**) Peak 1, retention time (RT) = 2.585 min; (**c**) Peak 2, RT = 3.236 min; (**d**) Peak 3, RT = 5.049 min; (**e**) Peak 4, RT = 5.788 min; (**f**) Peak 5, RT = 6.156 min; (**g**) Peak 6, RT = 6.572 min; (**h**) Peak 7, RT = 8.548 min; (**i**) Peak 8, RT = 12.748 min.

**Scheme 2:**
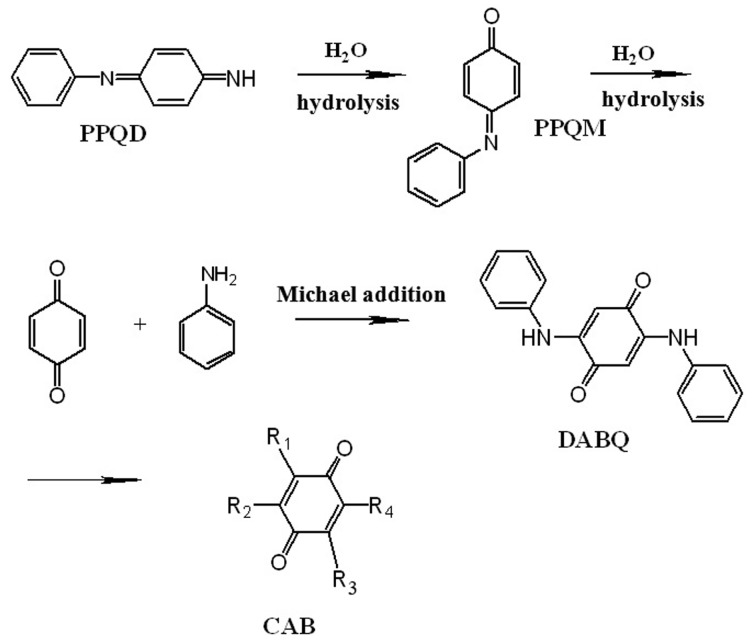
The formation process of the by-products in the used electrolyte solution, R${}_{1}$, R${}_{2}$, R${}_{3}$, R${}_{4}$ = H, N-phenylamino group, etc.

The composition of the n-octanol organic phase (ONOCT) and 2-octanone organic phase (O2OCT) obtained by extracting the 10th electrolyte solution was further investigated by HPLC-DAD, and the results are presented in Figure 3 and Figure 4. Figure 3a, the HPLC chromatogram of ONOCT, has nine chromatographic peaks. The UV-Vis spectra of the nine peaks are shown in Figure 3b–h, and the first seven chromatographic peaks from Peaks 1–7 correspond to HQ, BQ, aniline, *N*-phenyl-1,4-benzoquinone diimine (PBQD) [23,24], *N*-phenyl-1,4-benzoquinonemonoimine (PBQM) [23], *N*-phenyl-p-phenylene diamine (PPD) [24], 2,5-dianilino-p-benzoquinone (DABQ) [25], respectively. Peaks 8 and 9 belong to the CAB with larger molecular weight, which has a similar structure to DABQ [19]. The results of the HPLC test on ONOCT can be seen in Table 1. In Figure 4a, there are eight chromatographic peaks in the HPLC chromatogram of O2OCT. Most of them are the same as that of ONOCT. Comparing Figure 3a with Figure 4a, we can find that there was no aniline in O2OCT. Peak 7 of the ONOCT belonging to 2-octanone may cover the one peak of CAB, which is the same as Peak 8 in Figure 3a. PPD and PBQD represent the reduction state and oxidation state of the aniline dimmers, respectively. The results of the HPLC test on O2OCT can be seen in Table 2. The formation process of the by-products is shown in Scheme 2. As oxidation polymerization of aniline continues, 1,4-benzoquinone (BQ) is generated through imine hydrolysis, and then, it reacts with the amines through the addition reaction to generate co-oligomers of aniline and BQ (CAB) [25,26]. The ONOCT contains HQ, BQ, aniline dimers, CAB and aniline. The O2OCT contains HQ, BQ, aniline dimers and CAB. Thus, no aniline is detected in the O2OCT. Comprehensively analyzing the results of UV-Vis and HPLC-DAD, the main compositions of 10th electrolyte solution include aniline, HQ, BQ, PBQM, aniline dimers, CAB and acid. The concentration of aniline in the 10th electrolyte solution is equal to the concentration of aniline in the 2-octanone extracted aqueous phase (2OCT), which is about 0.118 M calculated from absorbance in 254.0 nm using the external standard method, as seen in Figure 2b. The fresh electrolyte solution was composed of 0.25 M aniline. About 47.2% of aniline remains in the waste electrolyte solution. Due to the difficulty of the complete elimination of aniline, recycling of electrolyte solution is necessary and important.

### 3.2. Influence of BQ in the Electrolyte Solution on PANI

**Figure 5:**
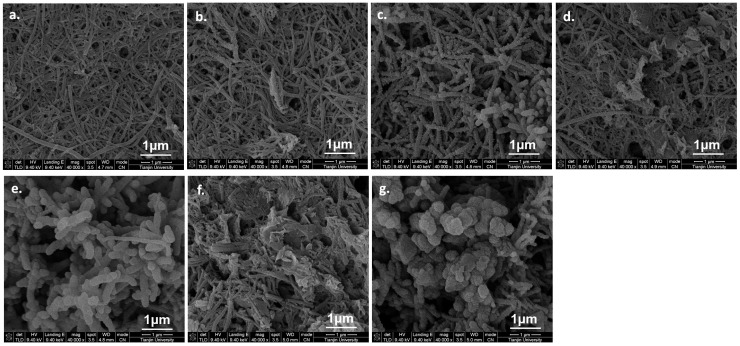
The SEM images of: (**a**) PANI obtained by electropolymerization in fresh electrolyte solution (P-Fresh); (**b**,**c**) PANI obtained by electropolymerization in electrolyte solution with the addition of 0.001 M BQ (P-BQ1); (**d**,**e**) PANI obtained by electropolymerization in fresh electrolyte solution with the addition of 0.005 M BQ(P-BQ2); and (**f**,**g**) PANI obtained by electropolymerization in fresh electrolyte solution with the addition of 0.01 M BQ (P-BQ3).

**Scheme 3:**
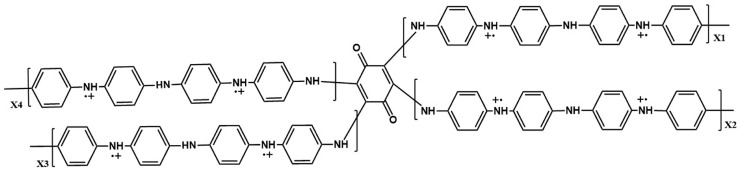
One possible cross-link structure of PANI.

As seen in Figure 5a, without adding BQ, the product P-Fresh presents a nanowire shape with diameters ranging from 50–130 nm and several microns in length. When BQ was added to the electrolyte solution, there was submicron rod-, nanosheet- and aggregated particle-shaped (non-nanowire shape) PANI appearing in the products. The amount of non-nanowire-shaped PANI increases with increasing the BQ concentration, as seen in Figure 5b–g. The difference in PANI morphology is due to the Michael addition reaction (Scheme 2) of amines and BQ [20]. The non-nanowire-shaped PANI is ascribed to the reaction of BQ with the amines of aniline or aniline oligomers to produce CAB. Then, the CAB can grow along the direction of the hydrogen bond and π-π* stacking, resulting in the self-assembly of nanosheets [19]. The non-nanowire-shaped PANI is also ascribed to the increasing cross-link degree of PANI due to the reaction of BQ with aniline or aniline oligomers, which changes the nucleation and growth and, finally, leads to the generation of submicron rods and aggregated particles. One possible cross-link structure of PANI is shown in Scheme 3.

As seen in Table 3, the specific capacitances of P-Fresh, P-BQ1, P-BQ2 and P-BQ3 are 576.6, 544.5, 524.7 and 499.8 F/g, calculated from CV tests, respectively. P-Fresh with the nanowire shape exhibits the highest specific capacitances because nanowires have a large specific surface area and can form three-dimensional porous networks, which facilitate ion mass transfer [27]. With the increase of the BQ concentration in the electrolyte solution, the specific capacitance of PANI decreases, which is ascribed to the decrease of the specific surface area and the passivation of ion mass transfer.

The BQ, HQ and CAB in the electrolyte solution participate in the polymerization of aniline, which results in the changes of the morphology of PANI, but also the purity and specific capacitance of PANI decrease.

### 3.3. The Morphology and Electrochemical Deterioration of PANI through the Long-Term Electrochemical Polymerization Process

**Figure 6:**
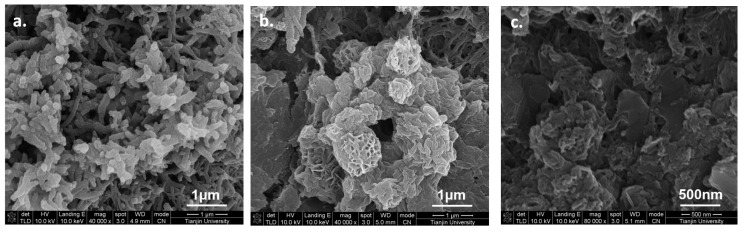
The SEM images of (**a**–**c**) PANI obtained by electropolymerization in the 10-th electrolyte solution (P-10).

**Figure 7:**
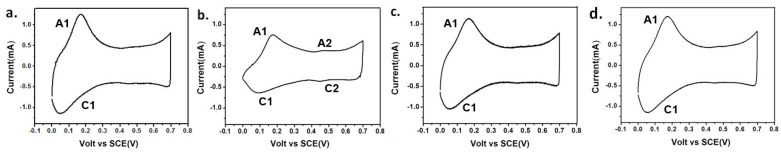
The CV curves of: (**a**) PANI obtained by electropolymerization in fresh electrolyte solution (P-Fresh); (**b**) PANI obtained by electropolymerization in the 10th electrolyte solution (P-10); (**c**) PANI obtained by electropolymerization in recycled electrolyte RNOCT (P-RNOCT); (**d**) PANI obtained by electropolymerization in recycled electrolyze 2OCT (P-R2OCT).

**Scheme 4:**
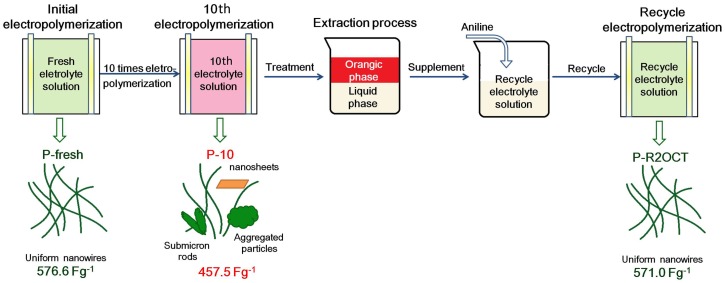
The diagram of the treatment and recycling procedure of the used electrolyte solution.

P-10 is obtained by electropolymerization in the 10th electrolyte solution, which is studied as the PANI prepared in the long-term electrochemical polymerization process. There are submicron rods, nanosheets and aggregated particles generated in P-10, as seen in Figure 6a–c, while the morphology of P-Fresh is comprised of nanowires. As seen in Figure 7a, the oxidation (A1 = 0.17 V vs. SCE) and corresponding reduction peaks (C1 = 0.04 V vs. SCE) belong to the reversible redox between the leucoemeraldine and emeraldine salt forms of polyaniline. Compared to P-Fresh, the CV curves of P-10 have extra oxidation (A2 = 0.48 V vs. SCE) and corresponding reduction peaks (C1 = 0.45 V vs. SCE) in 0.48 V vs. SCE, which belongs to oxidation process of the HQ to BQ structure in the CAB [28], as seen in Figure 7a,b. The specific capacitance of P-Fresh is 576.6 F/g, and the specific capacitance of P-10 is 457.5 F/g, calculated from the CV test, as seen in Table 3. The morphology changes and specific capacitance decrease of P-10 (Scheme 4) are ascribed to the accumulation of the by-products HQ, BQ and CAB through electropolymerization, as discussed in Section 3.2. In order to recover the morphology and specific capacitance of PANI, selective removal of the by-products HQ, BQ and CAB is needed.

### 3.4. The Treatment and Recycling of Used Electrolyte Solution

**Figure 8:**
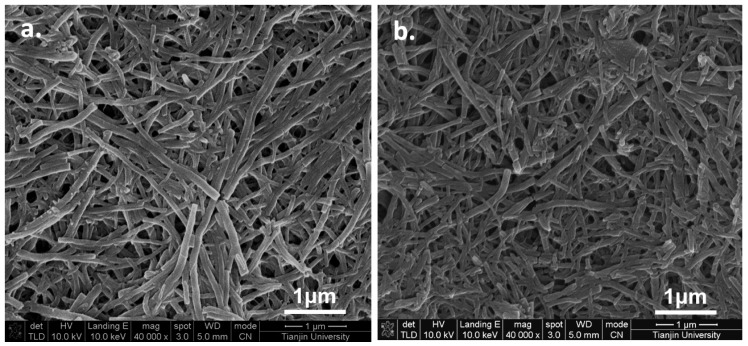
The SEM images: (**a**) PANI obtained by electropolymerization in recycled electrolyte solution NOCT (P-RNOCT); and (**b**) PANI obtained by electropolymerization in recycled electrolyte solution NOCT (P-R2OCT).

In this paper, the extraction process is chosen as the treatment of the used electrolyte solution, in order to selectively remove the by-products HQ, BQ and CAB. The n-octanol and 2-octanone were used as extraction solvents. According to the result of HPLC analysis in Section 3.1, the ONOCT contains HQ, BQ, aniline dimers, CAB and aniline; the O2OCT contains HQ, BQ, aniline dimers and CAB. A small amount of aniline is extracted in the n-octanol, so the extraction solvent 2-octanone is more selective for the treatment. The composition of NOCT and 2OCT was investigated by UV-Vis. The absorption peak in the UV-Vis spectrum of NOCT and 2OCT is 254.0 nm, which belongs to protonated aniline, as seen in Figure 2a,b. After the extraction of the 10th electrolyte solution by n-octanol or 2-octanone, the by-products HQ, BQ and CAB are selectively removed from the electrolyte solution. After that, the recycled electrolyte solution is obtained by increasing the concentration of aniline in the treated electrolyte solution to 0.25 M, respectively. The electrochemical polymerization process was conducted. The morphology and specific capacitance of PANI are recovered through the recycled electrolyte solution. The morphology of P-RNOCT and P-R2OCT is comprised of nanowires with 50–130 nm diameters and several microns in length, which was about the same as that of P-Fresh, as seen in Figure 5a and Figure 8a,b. The CV curves of P-RNOCT and P-R2OCT have the same redox peaks (A1 and C1) as the CV curves of P-Fresh, as seen in Figure 7a,c,d. What is more, the specific capacitance of P-RNOCT and P-R2OCT is 555.8 F/g and 571.0 F/g, as shown in Table 3, which was about the same as that of P-Fresh (576.6 F/g). The flow and result of the treatment and recycled used electrolyte solution can be seen in Scheme 4.

## 4. Conclusions

The electrochemical polymerization process is a potential method for PANI nanowire preparation. In this research, the PANI obtained by the polymerization in fresh electrolyte solution (P-Fresh) has a uniform nanowire shape and high specific capacitance (576.6 F/g). However, as the electrochemical polymerization of aniline goes on, the by-products (BQ, HQ and CAB) are generated and accumulate in the electrolyte solution The BQ, HQ and CAB in the electrolyte solution participate in the polymerization of aniline, resulting in the changes of PANI morphology, as well as the decrease of the purity and specific capacitance. There are submicron rod-, nanosheet- and aggregated particle-shaped PANI appearing in the P-10, which is obtained by polymerization of the used electrolyte solution. The specific capacitance of P-10 is decreased to 457.5 F/g. In order to recycle the waste electrolyte solution, n-octanol and 2-octanone are chosen as the extraction solvent to selectively remove the HQ, BQ and CAB from the used electrolyte solution. PANI produced in the recycled electrolyte solution (P-R2OCT) has a uniform nanowire shape and high specific capacitance (571.0 F/g), which is similar to P-Fresh. Through the recycling of the waste electrolyte solution, the process of the electrochemical production of PANI nanowires could be more environmentally friendly and lower in cost. This recycled extraction method might also be used in the recycling of waste water by low-yield chemical methods to produce PANI nanowires, such as rapid mixing methods [27,29].

## Figures and Tables

**Table 1:** The result of HPLC tests on the n-octanol organic phase (ONOCT).

Peaks Number	Retention Time (min)	Absorption Peaks (nm)	Compound
Peak 1	2.597	225.0, 293.6	HQ
Peak 2	3.250	245.1	BQ
Peak 3	3.795	234.4, 285.3	aniline
Peak 4	5.078	259.3, 508.8	PBQD
Peak 5	5.814	264.8, 285.0, 445.8	PBQM
Peak 6	6.190	283.0	PPD
Peak 7	6.572	265.2, 383.4	DABQ
Peak 8	9.115	264.8, 367.4	CAB
peak 9	12.901	265.0, 383.0	CAB

**Table 2:** The result of HPLC tests on the 2-octanone organic phase (O2OCT).

Peaks Number	Retention Time (min)	Absorption Peaks (nm)	Compound
Peak 1	2.597	225.0, 293.6	HQ
Peak 2	3.250	245.1	BQ
Peak 3	5.049	259.3, 508.8	PBQD
Peak 4	5.788	264.8, 285.0, 445.8	PBQM
Peak 5	6.156	283.0	PPD
Peak 6	6.572	265.2, 383.4	DABQ
Peak 7	8.648	275.8	2-octanone
Peak 8	12.748	265.0, 383.0	CAB

**Table 3:** The specific capacitance of PANI.

PANI Sample	Electrolyte Solution	Specific Capacitance (F/g)
P-Fresh	Fresh electrolyte solution	576.6
P-10	10th electrolyte solution	457.5
P-RNOCT	RNOCT	555.8
P-R2OCT	R2OCT	571.0
P-BQ1	Fresh electrolyte solution 0.001 M BQ	544.5
P-BQ2	Fresh electrolyte solution 0.005 M BQ	524.7
P-BQ3	Fresh electrolyte solution 0.010 M BQ	499.8

## Abbreviations

The following abbreviations are used in this manuscript:

A	ampere
V	volt
g	gram
PANI	polyaniline
HPLC-DAD	high performance liquid chromatography coupled with diode array detection
UV-Vis	ultraviolet and visible
HQ	p-hydroquinone
BQ	p-benzoquinone
CAB	co-oligomers of aniline and p-benzoquinone
ACN	acetonitrile
SS	stainless steel
SCE	saturated calomel electrode
CV	cyclic voltammetry
SEM	scanning electron microscope
n-th electrolyte solution	electrolyte solution after n repetitions of the electropolymerization of aniline
10th electrolyte solution	electrolyte solution after 10 repetitions of the electropolymerization of aniline
NOCT	aqueous phase extracted by n-octanol, after 13 extractions of the 10th electrolyte
2OCT	aqueous phase extracted by 2-octanone, after 13 extractions of the 10th electrolyte
ONOCT	n-octanol organic phase obtained by extracting the 10th electrolyte solution
O2OCT	2-octanone organic phase obtained by extracting the 10th electrolyte solution
RNOCT	recycled electrolyze NOCT
R2OCT	recycled electrolyze 2OCT
P-Fresh	the PANI obtained by electropolymerization in fresh electrolyte solution
P-10	the PANI obtained by electropolymerization in solution 10th electrolyte solution
P-RNOCT	the PANI obtained by electropolymerization in RNOCT
P-R2OCT	the PANI obtained by electropolymerization in R2OCT
P-BQ1	the PANI obtained by fresh electrolyte solution with the addition of 0.001 M BQ
P-BQ2	the PANI obtained by fresh electrolyte solution with the addition of 0.005 M BQ
P-BQ3	the PANI obtained by fresh electrolyte solution with the addition of 0.01 M BQ
PBQD	*N*-phenyl-1,4-benzoquinone diimine
PBQM	*N*-phenyl-1,4-benzoquinonemonoimine
PPD	*N*-phenyl-p-phenylene diamine
DABQ	2,5-dianilino-p-benzoquinone

## References

[1] Sutar D.S., Padma N., Aswal D.K., Deshpande S.K., Gupta S.K., Yakhmi J.V. (2007). Preparation of nanofibrous polyaniline films and their application as ammonia gas sensor. Sens. Actuators.

[2] Chen J., Yang J., Yan X., Xue Q. (2010). NH_3_ and HCl sensing characteristics of polyaniline nanofibers deposited on commercial ceramic substrates using interfacial polymerization. Synth. Met..

[3] Zhang H., Li H., Zhang F., Wang J., Wang Z., Wang S. (2008). Polyaniline nanofibers prepared by a facile electrochemical approach and their supercapacitor performance. J. Mater. Res..

[4] Sivakkumar S.R., Oh J.-S., Kim D.-W. (2006). Polyaniline nanofibres as a cathode material for rechargeable lithium-polymer cells assembled with gel polymer eletrolyte solution. J. Power Sources.

[5] Gilja V., Novaković K., Travas-Sejdic J., Hrnjak-Murgić Z., Kraljić Roković M., Žic M. (2017). Stability and Synergistic Effect of Polyaniline/TiO_2_ Photocatalysts in Degradation of Azo Dye in Wastewater. Nanomaterials.

[6] Song E., Choi J.-W. (2013). Conducting Polyaniline Nanowire and Its Applications in Chemiresistive Sensing. Nanomaterials.

[7] Gupta V.K., Nayak A., Agarwal S. (2012). Performance evaluation and application of oxygen enriched waste rubber tire adsorbent for the removal of hazardous aniline derivatives from waste water. Chem. Eng. J..

[8] Golovashin V.L., Lazarev S.I., Mamontov V.V. (2005). Kinetic characteristics of reverse-osmosis separation of an aqueous solution of aniline in a flat-frame apparatus. Russ. J. Appl. Chem..

[9] Zhou L., Hu J., Zhong H., Li X. (2012). Study of phenol removal using fluidized-bed Fenton process. Chem. Eng. Res. Des..

[10] Masomboon N., Ratanatamskul C., Lu M.-C. (2009). Chemical Oxidation of 2,6-Dimethylaniline in the Fenton Process. Environ. Sci. Technol..

[11] Yang B., Ying G., Zhao J.L., Liu S., Zhou L.J., Chen F. (2012). Removal of selected endocrine disrupting chemicals (EDCs) and pharmaceuticals and personal care products (PPCPs) during ferrate(VI) treatment of secondary wastewater effluents. Water Res..

[12] Pacheco M.J., Santos V., Ciriaco L., Lopes A. (2011). Electrochemical degradation of aromatic amines on BDD electrodes. J. Hazard. Mater..

[13] Masomboon N., Ratanatamskul C., Lu M.-C. (2010). Chemical oxidation of 2,6-dimethylaniline by electrochemically generated Fenton’s reagent. J. Hazard. Mater..

[14] Karim Z., Husain Q. (2009). Redox-mediated oxidation and removal of aromatic amines from polluted water by partially purified bitter gourd (*Momordica charantia*) peroxidase. Int. Biodeterior. Biodegrad..

[15] Zhang S., Li A., Cui D., Yang J., Ma F. (2011). Performance of enhanced biological SBR process for aniline treatment by mycelial pellet as biomass carrier. Bioresour. Technol..

[16] Liu J., Guan J., Lu M., Kan Q., Li Z. (2012). Hemoglobin immobilized with modified “fish-in-net” approach for the catalytic removal of aniline. J. Hazard. Mater..

[17] Sun R., Wang Y., Ni Y., Kokot S. (2014). Spectrophotometric analysis of phenols, which involves a hemin-graphene hybrid nanoparticles with peroxidase-like activity. J. Hazard. Mater..

[18] Sapurina I., Stejskal J. (2008). The mechanism of the oxidative polymerization of aniline and the formation of supramolecular polyaniline structures. Polym. Int..

[19] Zhao Y., Tomsik E., Wang J., Moravkova Z., Zhigunov A., Stejskal J., Trchova M. (2013). Self-Assembly of Aniline Oligomers. CHEM-ASIAN J..

[20] Silva C.H.B., Ferreira D.C., Constantino V.R.L., Temperini M.L.A. (2011). Characterization of the products of aniline peroxydisulfate oligo/polymerization in media with different pH by resonance Raman spectroscopy at 413.1 and 1064 nm excitation wavelengths. J. Raman Spectrosc..

[21] Johnson B.J., Park S.M. (1996). Electrochemistry of Conductive Polymers: XX. Early Stages of Aniline Polymerization Studied by Spectroelectrochemical and Rotating Ring Disk Electrode Techniques. J. Raman Spectrosc..

[22] Hong S.Y., Jung Y.M., Kim S.B., Park S.M. (2005). Electrochemistry of Conductive Polymers. 34. Two-Dimensional Correlation Analysis of Real-Time Spectroelectrochemical Data for Aniline Polymerization. J. Phys. Chem. B.

[23] Balón M., Guardado P., Carmona C., Hidalgo J., Munoz M.A. (1993). Influence of the acidity on the kinetics of diphenylamine oxidation by peroxodisulfate anions. Can. J. Chem..

[24] Nayaki S.K., Swaminathan M. (2006). Excited state solvatochromic and prototropic behaviour of 4-aminodiphenylamine and 4,4’-diaminodiphenylamine—A comparative study by electronic spectra. Spectrochim. Acta Part A Spectrosc..

[25] Stejskal J., Bober P., Trchova M., Horsky J., Pilar J., Walterova Z. (2014). The oxidation of aniline with p-benzoquinone and its impact on the preparation of the conducting polymer, polyaniline. Synth. Met..

[26] Tandon V.K., Maurya H.K. (2009). ‘On water’: Unprecedented nucleophilic substitution and addition reactions with 1,4-quinones in aqueous suspension. Tetrahedron Lett..

[27] Zhao Y., Wei H., Arowo M., Yan X., Wu W., Chen J., Wang Y., Guo Z. (2015). Electrochemical energy storage by polyaniline nanofibers: High gravity assisted oxidative polymerization vs. rapid mixing chemical oxidative polymerization. PCCP.

[28] Milczarek G., Inganas O. (2012). Renewable Cathode Materials from Biopolymer/Conjugated Polymer Interpenetrating Networks. Science.

[29] Chiou N.R., Epstein A.J. (2005). A simple approach to control the growth of polyaniline nanofibers. Synth. Met..

